# Unilateral Hormonally Active Testicular Sertoli Cell Tumour and Feminization Syndrome in a Bernese Mountain Dog With Normally Descended Testes

**DOI:** 10.1111/rda.70323

**Published:** 2026-11-02

**Authors:** Ariu Federica, Burrai Giovanni Pietro, Cubeddu Francesca, Masala Gerolamo, Moalli Marzia, Antuofermo Elisabetta, Corda Francesca, Pinna Parpaglia Maria Luisa, Corda Andrea

**Affiliations:** ^1^ Department of Veterinary Medicine University of Sassari Sassari Italy

**Keywords:** feminization syndrome, hyperestrogenism, preputial cytology, Sertoli cell tumour

## Abstract

To describe the clinical presentation, diagnostic workup and successful outcome of a hormonally active testicular Sertoli cell tumour (SCT) in a non‐cryptorchid Bernese Mountain Dog. A 10‐year‐old intact male dog was evaluated with a history of progressive alopecia, gynaecomastia and haematuria. Diagnostic investigations included complete blood count, serum biochemistry, oestradiol assay, abdominal and testicular ultrasonography, preputial cytology and histopathological examination following bilateral orchiectomy. Clinical signs included bilaterally symmetrical alopecia, hyperpigmentation and a pendulous prepuce. Laboratory findings revealed mild non‐regenerative anaemia and severe hyperestrogenism (oestradiol: 119 pg/mL). Preputial cytology showed a predominance of superficial epithelial cells (> 80%), supporting the state of hyperestrogenism. Ultrasonography identified a large, vascularized left testicular mass and cystic prostatic changes. Histopathology confirmed an SCT. Following surgery, clinical signs and laboratory abnormalities resolved completely within 9 months. This case emphasizes that SCTs should be a differential diagnosis for feminization syndrome in older dogs even without cryptorchidism. It also highlights preputial cytology as a rapid and effective minimally invasive tool for the preliminary identification of hyperestrogenism.

## 1 Introduction

Testicular Sertoli cell tumours (SCTs) accounted for 14%–33% of diagnosed testicular tumours in dogs (Hayes and Pendergrass 1976; Hohšteter et al. 2014; Liao et al. 2009; Manuali et al. 2020). Like other testicular tumours, SCTs are most common in cryptorchid testes, and dogs with inguinal hernias are reported to have a higher tumour risk (Hayes et al. 1985). Usually, SCTs are slow‐growing and non‐invasive tumours with a metastasis rate of less than 10% (Subapriya et al. 2025). The feminizing syndrome associated with SCT is linked to hyperestrogenism and develops in approximately 57% of dogs with SCTs (Mischke et al. 2002; Weaver 1983). It is characterized by non‐pruritic, bilaterally symmetrical alopecia, hyperpigmentation, gynaecomastia and a pendulous, oedematous prepuce (da Silva Flores et al. 2023; Fadok et al. 1986; Quartuccio et al. 2012). Although feminization syndrome is classical in cryptorchid cases due to the higher prevalence and neoplastic transformation rate of retained gonads, it can also manifest in dogs with normally descended testes. Oestrogen‐induced myelotoxicity, characterized by bone marrow hypoplasia, has also been reported in association with SCTs (Choi et al. 2008; Marshall 2018; Paepe et al. 2016; Post and Kilborn 1987; Sanpera et al. 2002; Sherding et al. 1981; Sontas et al. 2009; Withers et al. 2016). In clinical practice, the diagnostic approach to canine testicular tumours involves physical examination, testicular ultrasonography and histological confirmation, while differential diagnosis includes other primary testicular neoplasms, particularly leydigomas and seminomas.

This report describes a clinical case of testicular SCT associated with feminization syndrome in a 10‐year‐old male dog with normally descended testes.

## 2 Case History

A 10‐year, 6‐month‐old, intact, non‐cryptorchid, male Bernese Mountain Dog, weighing 42.5 kg, was referred to the Veterinary Teaching Hospital of the University of Sassari for clinical evaluation because of bilateral, non‐pruritic alopecia involving the abdomen, lumbar region, thorax and perianal area. The owners also reported intermittent preputial haemorrhagic discharge and haematuria occurring periodically over the previous few months, as well as reluctance to climb stairs and mild lethargy, which they attributed to the dog's age. No other behavioural changes were reported. The dog was regularly vaccinated, routinely dewormed and treated for ectoparasites, fed a commercial dry diet and had indoor and outdoor access.

## 3 Clinical and Laboratory Findings

Physical examination revealed normal general health parameters, including a rectal temperature of 38.1°C, pulse rate of 118 bpm, respiratory rate of 19 breaths/min and pink, moist mucous membranes with a capillary refill time < 2 s and a body condition score (BCS) of 2/5. Specific clinical findings included a dry, dull, discoloured, woolly‐type hair coat with extensive symmetrical, non‐pruritic alopecia (Figure 1A) and hyperpigmented skin. The prepuce was oedematous and pendulous (Figure 1D), gynaecomastia (Figure 1C) was present, papules were observed along the mammary line and penile region, and the left testicle was larger than the right. The dog underwent blood and urine analysis, preputial cytology and abdominal ultrasonography.

**FIGURE 1:**
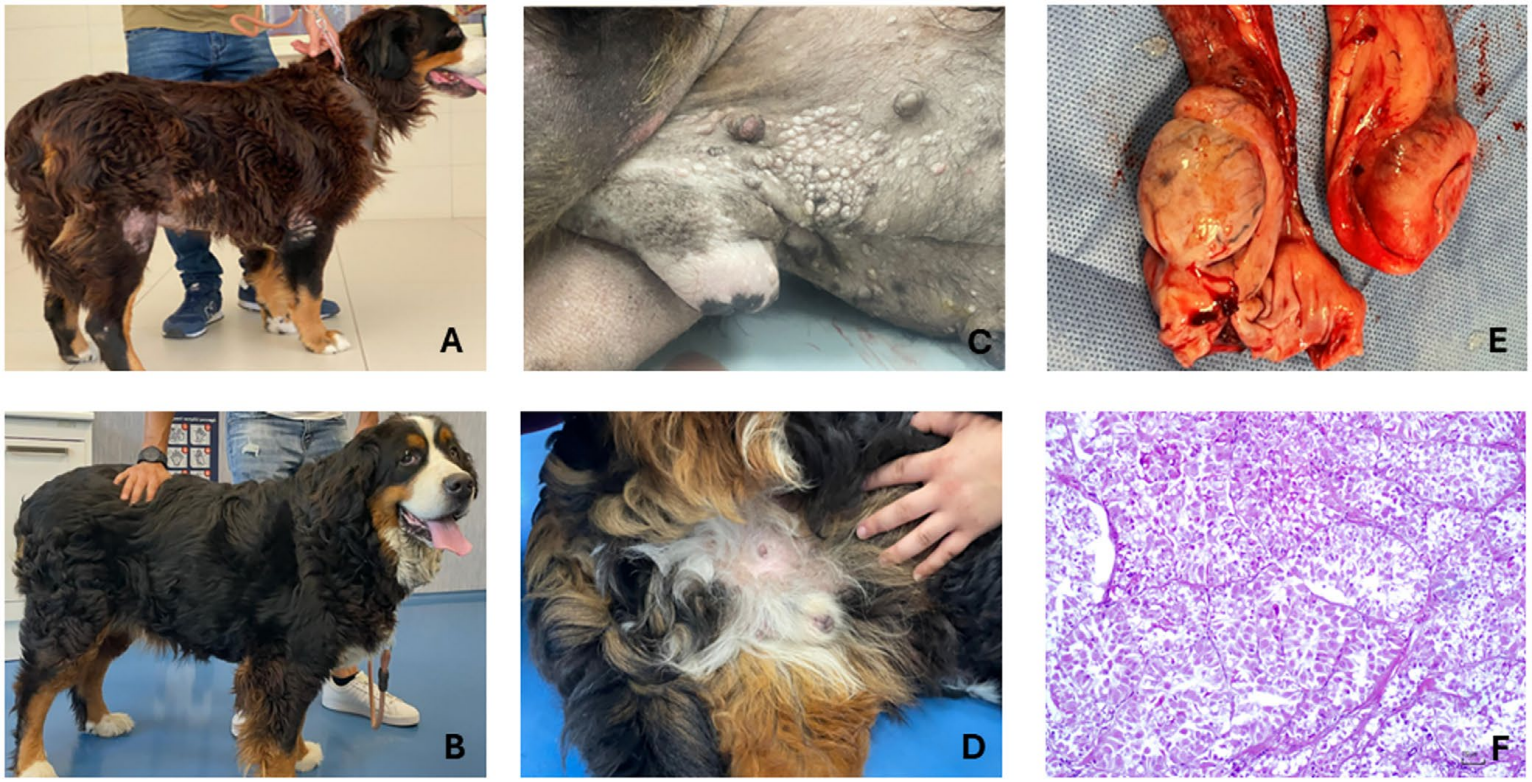
Clinical, gross and histological findings of a Sertoli cell tumour (SCT) in a 10‐year‐old Bernese Mountain Dog. (A, B) Clinical presentation at admission showing severe, bilaterally symmetrical alopecia of the trunk and hindquarters, hyperpigmentation and gynaecomastia (top left). Follow‐up at 9 months post‐castration (top right) demonstrating complete hair regrowth and resolution of skin lesions. (C, D) Detail of the pendulous and oedematous prepuce (middle left) and gynaecomastia with enlarged nipples (middle right), characteristic signs of feminization syndrome. (E) Gross appearance of the testes after bilateral orchiectomy. The left testis is markedly enlarged by a multinodular, firm, whitish mass that replaces the normal parenchyma. (F) Histopathological section of the testicular mass (haematoxylin and eosin, 20×). The neoplastic proliferation is characterized by tubular structures lined by elongated, palisading Sertoli cells with vacuolated cytoplasm, confirming the diagnosis of Sertoli cell tumour.

Complete blood count revealed a mild normocytic, normochromic, non‐regenerative anaemia (Table 1). Serum biochemical analysis showed a mild increase in aspartate aminotransferase (AST 85 IU/L, reference intervals [RIs] 17–52 IU/L) and increased total protein concentration (TP 7.87 g/dL, RI 5.1–7.5 g/dL) due to hyperglobulinemia (GLOB 5.21 g/dL, RI 2.4–4.3 g/dL). Serum protein electrophoresis revealed a relative decrease in albumin (ALB 38.2%, RI 45.5%–66.2%), increased beta (β‐GLOB 26.5%, RI 12.8–23.7) and gamma globulin (γ‐GLOB 22.1%, RI 5.1%–17.8%) fractions. In addition, serum biochemistry revealed an elevation in creatine kinase (CPK 895 U/L) and hyperphosphatemia (11.6 mg/dL) with an increased anion gap (34.0), while renal function parameters (urea and creatinine) remained within normal RIs. The main serum biochemical and electrophoretic findings are summarized in Table 2.

**TABLE 1:** Complete blood count (CBC) of the Bernese Mountain Dog at presentation.

Parameter	Patient value	Reference interval	Status/note
Red blood cells (RBC)	5.98 × 10^6^/μL	5.90–8.70	Normal
Haemoglobin (HGB)	13.00 g/dL	13.90–20.40	**Low**
Haematocrit (HCT)	40.50%	43.20–63.20	**Low**
Mean corpuscular volume (MCV)	67.70 fL	65.10–82.10	Normal
Mean corpuscular HGB (MCH)	21.70 pg	21.40–25.60	Normal
MCH concentration (MCHC)	32.10 g/dL	28.80–35.20	Normal
Red cell distribution width (RDW)	17.10%	12.10–15.70	**High** (Anisocytosis +++)
Reticulocytes	65,182/μL (1.09%)	11,504–147,178	Non‐regenerative
White blood cells (WBC)	11.35 × 10^3^/μL	5.60–14.00	Normal
Segmented neutrophils	7673/μL (67.6%)	3100–10,400	Normal
Lymphocytes	2145/μL (18.9%)	1100–4300	Normal
Monocytes	885/μL (7.8%)	100–900	Normal
Eosinophils	636/μL (5.6%)	100–1200	Normal
Basophils	11/μL (0.1%)	0–100	Normal
Platelets (PLT)	351 × 10^3^/μL	110–475	Normal (adequate)

*Note:* Bold text/values indicate results outside the reference interval.

Abbreviations: CBC, complete blood count; HCT, haematocrit; HGB, haemoglobin; MCH, mean corpuscular haemoglobin; MCHC, mean corpuscular haemoglobin concentration; MCV, mean corpuscular volume; PLT, platelets; RBC, red blood cells; RDW, red cell distribution width; WBC, white blood cells.

**TABLE 2:** Serum biochemistry profile of the Bernese Mountain Dog at presentation.

Parameter	Patient value	Reference interval	Status/note
Creatine kinase (CPK)	**895 U/L**	35–200	**High**
Aspartate aminotransferase (AST)	**85 U/L**	17–52	**High**
Alanine aminotransferase (ALT)	56 U/L	19–76	Normal
Alkaline phosphatase (ALP)	67 U/L	12–134	Normal
Gamma‐glutamyl transferase (GGT)	8 U/L	2–9	Normal
Total protein	**7.87 g/dL**	5.10–7.50	**High**
Albumin	2.66 g/dL	2.40–3.70	Normal
Globulins	**5.21 g/dL**	2.40–4.30	**High**
Albumin/globulin ratio (A/G)	**0.51**	0.60–1.30	**Low**
Urea	34 mg/dL	18–50	Normal
Creatinine	1.12 mg/dL	0.60–1.40	Normal
Cholesterol	233 mg/dL	131–332	Normal
Triglycerides	39 mg/dL	32–114	Normal
Calcium	10.1 mg/dL	9.1–11.5	Normal
Corrected calcium	10.9 mg/dL	9.9–12.1	Normal
Phosphorus	**11.6 mg/dL**	2.5–5.5	**High**
Calcium × phosphorus product	**125.9**	< 60.0	**High**
Sodium	149 mEq/L	143–153	Normal
Potassium	5.5 mEq/L	3.9–5.7	Normal
Chloride	**103 mEq/L**	108–119	**Low**
Bicarbonate $\left({\mathrm{HCO}}_{3}^{-}\right)$	17.9 mEq/L	17.0–24.0	Normal
Anion gap	**34.0**	15.0–25.0	**High**
Iron (total)	98 μg/dL	80–214	Normal

*Note:* Bold text/values indicate results outside the reference interval.

Abbreviations: A/G, albumin/globulin ratio; ALP, alkaline phosphatase; ALT, alanine aminotransferase; AST, aspartate aminotransferase; CPK, creatine kinase; GGT, gamma‐glutamyl transferase; ${\mathrm{HCO}}_{3}^{-}$, bicarbonate.

The serum oestradiol concentration was 119.0 pg/mL, markedly exceeding the reference limit of < 15 pg/mL (Mischke et al. 2002).

The enzyme‐linked immunosorbent assay (ELISA) test for leishmaniosis was negative. Serum cholesterol and triglyceride concentrations, as well as basal and urinary cortisol levels and thyroid hormone concentrations, were within laboratory RIs. Urinalysis performed on a free‐catch urine sample revealed increased urine pH, haematuria and proteinuria with a urine protein‐to‐creatinine ratio of 2.91.

Cytological examination of the preputial smear, performed as previously described (Dreimanis et al. 2012), showed high cellularity characterized predominantly by superficial squamous cells (> 80% anucleated or pyknotic). Parabasal cells, intermediate cells and neutrophils were rarely observed (Figure 2).

**FIGURE 2:**
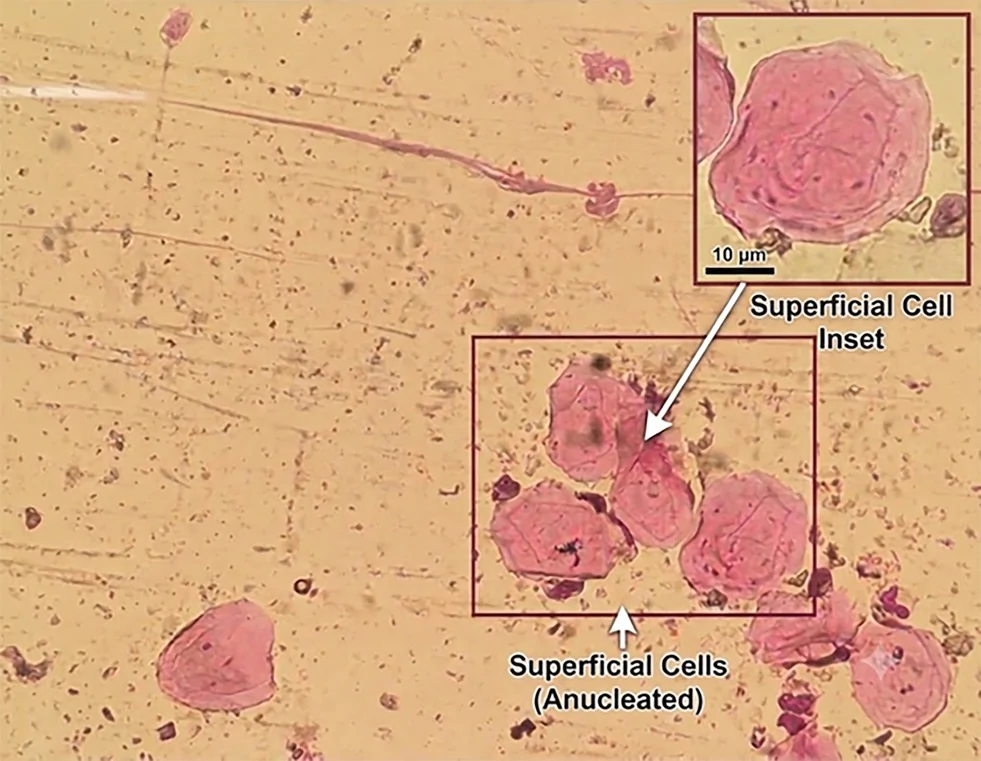
Preputial cytology smear showing high cellularity with a marked predominance (> 80%) of superficial squamous epithelial cells (anucleated or pyknotic), characteristic of estrogenic stimulation/hyperestrogenism (20× magnification; inset at 40× magnification).

Abdominal ultrasonography revealed symmetrical prostatomegaly, with a heterogeneous prostatic parenchymal echotexture and the presence of multiple irregularly shaped cystic structures that did not protrude from the organ surface (Figure 3). The left testicle was enlarged (approximately 2.7 × 3.5 cm) and showed a heterogeneous echotexture, irregular shape and altered internal architecture, with marked vascularization on colour Doppler examination (Figure 4). The right testicle had a normal shape, echogenicity and echotexture, but was subjectively reduced in size (approximately 2 × 1 cm) (Figure 5). Left medial iliac lymphadenomegaly was observed (approximately 4.1 cm × 1.4 cm); the lymph node had a fusiform shape and normal echogenicity. No other clinically relevant ultrasonographic abnormalities were detected.

**FIGURE 3:**
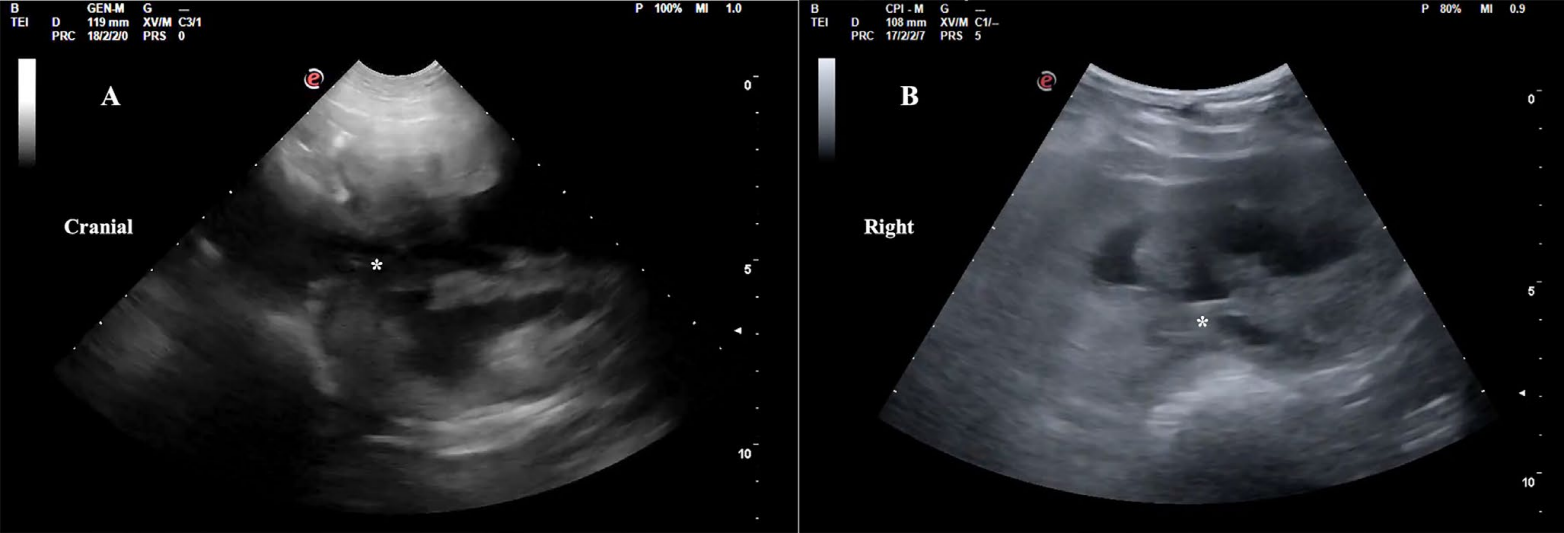
B‐mode images of the prostate in longitudinal (A) and transverse (B) sections. The organ appears enlarged, with large, irregularly shaped cystic structures within the parenchyma, which shows a heterogeneous echotexture.

**FIGURE 4:**
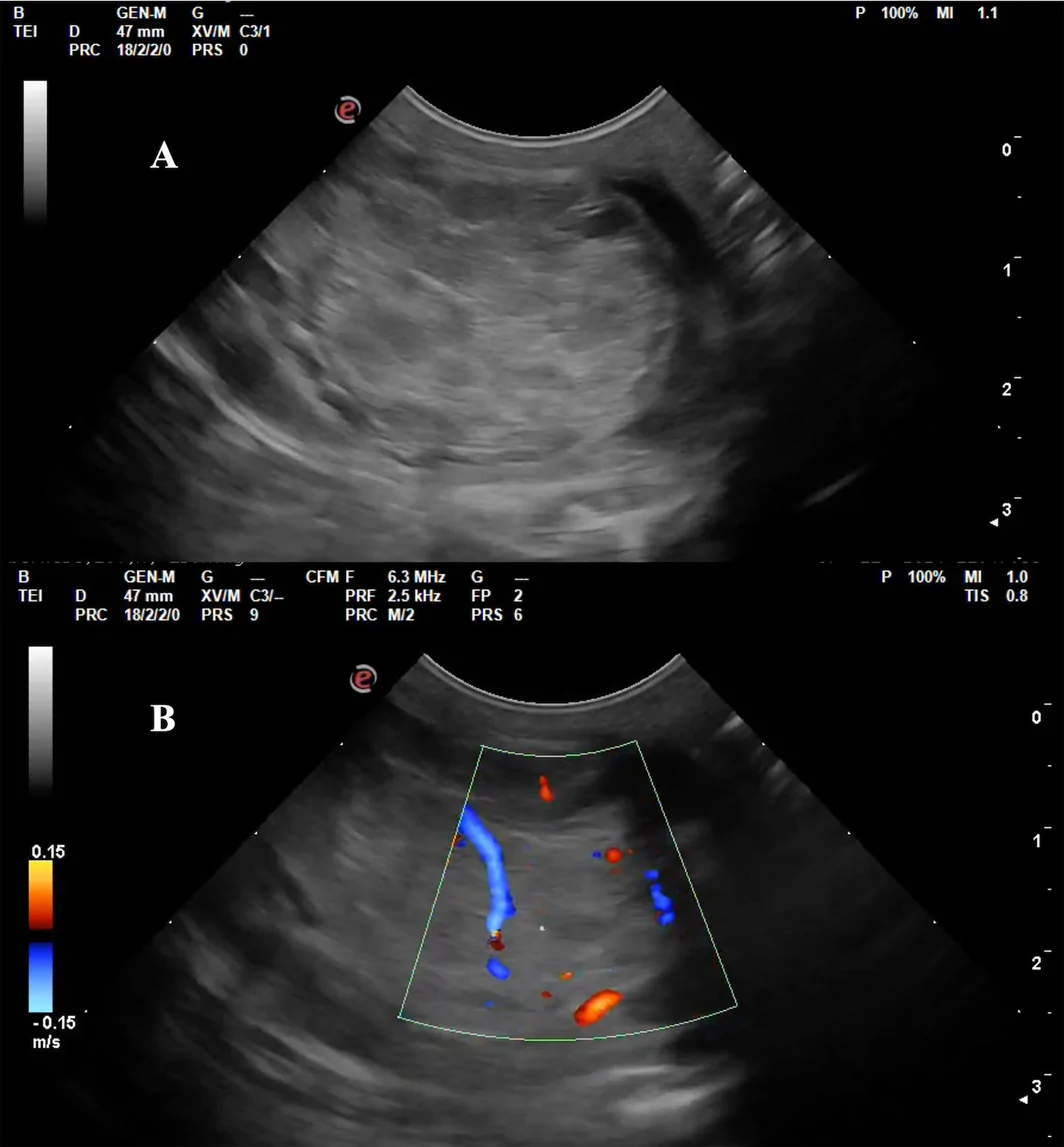
B‐mode (A) and colour Doppler (B) ultrasound images of the left testicle. The organ is enlarged, with irregular margins and heterogeneous echotexture (A). Colour Doppler examination revealed marked vascularization of the parenchyma.

**FIGURE 5:**
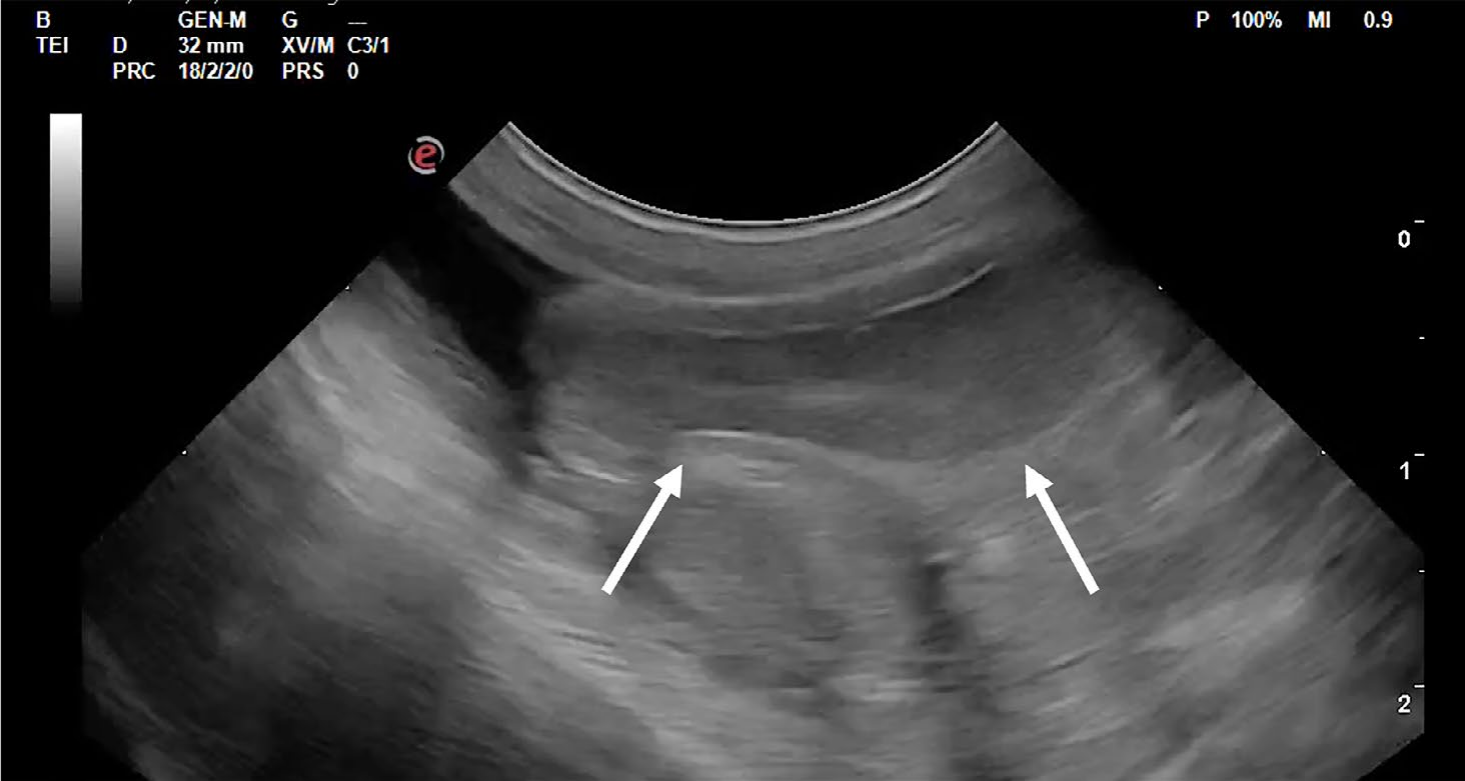
B‐mode image of a longitudinal section of the right testicle (arrows), showing a reduced‐size organ with preserved normal architecture, echotexture and echogenicity.

Ultrasound‐guided aspiration of the fluid contained within the prostatic cysts was performed, and the collected samples were subsequently submitted for bacteriological culture. In addition, fine‐needle aspiration cytology of the left medial iliac lymph node was performed. Bacteriological culture was negative, and lymph node cytology was consistent with reactive lymphoid hyperplasia.

Based on clinical laboratory and imaging diagnostic findings, feminization syndrome secondary to a unilateral testicular SCT was suspected, and orchiectomy with histopathological examination was recommended.

The dog was premedicated with medetomidine (2 μg/kg; Sedastart 0.1% Ecuphar S.r.l., Milano, Italy), butorphanol 0.2 mg/kg (Nargesic 1% ACME S.r.l., Reggio Emilia, Italy) and maropitant 1 mg/kg (Vetemex 1% Virbac S.r.l., Milano, Italy).

Anaesthesia was induced with propofol IV 2 mg/kg (Proposure 1%, Merial Italia, S.p.a., Milan, Italy) and maintained with sevoflurane (2%–2.2%; 0.9–1 MAC) (SevoFlo Zoetis Belgium SA, Louvain‐la‐Neuve, Belgium) in oxygen.

A complete scrotal ablation with bilateral orchiectomy was performed. Both testes and the scrotal tissue were submitted for histological examination.

## 4 Pathological Findings

On gross examination, a marked difference in the size and consistency of the testes was noted (Figure 1E). The left testicle appeared significantly enlarged (27.1 mm × 35.2 mm) and had a firm, irregular consistency, unlike the right testicle, which measured 15.6 mm × 23.1 mm and had a regular consistency. On incision, the mass of the left testicle showed a whitish colour and a homogeneous appearance, with some areas of haemorrhage and focal necrosis. The right testicle, however, appeared normal. Histological examination of the left testicle revealed a solid, multifocal and infiltrating neoplastic proliferation of tubular supporting epithelial cells, consistent with an SCT (Figure 1F). The neoplastic cells were arranged in cords, nests and occasional tubular structures, supported by a fine fibrovascular stroma. Moderate cellular and nuclear atypia was observed, with low mitotic activity (< 1 mitosis per high‐power field). Furthermore, extensive areas of coagulative necrosis were identified. The testicular parenchyma adjacent to the tumour showed severe germinal atrophy, with a marked reduction or absence of spermatogonia in the seminiferous tubules. The right testicle showed no significant changes.

Postoperative recovery was uneventful. Over the following months, cutaneous alterations, gynaecomastia, oedema and pendulous prepuce progressively resolved with complete coat regrowth, and the owners reported increased vitality and resolution of preputial bleeding. At 5‐ and 9‐month follow‐ups, the dog was in excellent general condition, with normal clinical examination and laboratory findings (Figure 1B). Occasional mild urinary leakage was reported by the owner. Overall, the outcome was favourable with a good long‐term prognosis.

## 5 Discussion

This case report describes feminization syndrome in an intact male dog with a left testicular SCT. SCTs are hormonally active tumours that originate from gonadostromal tissue within the testes and produce oestrogens and inhibin (Peters et al. 2000).

Although testicular SCTs are more common in cryptorchid dogs, they can also occur in scrotal testes. In dogs with normally descended testes, age is a major risk factor, with reported mean ages of 7.8 years for abdominal, 8.8 years for inguinal and 11 years for scrotal testes (Reif and Brodey 1969; Soto‐Heras et al. 2024). This consideration highlights the importance of including testicular palpation as part of the routine clinical examination, especially in older dogs, and even more so in those presenting with diffuse cutaneous changes.

The Bernese Mountain Dog is not listed among the breeds with the highest incidence of testicular tumours; however, purebred dogs overall are more frequently diagnosed with testicular tumours than mixed‐breed dogs (Ngo et al. 2025).

The feminization syndrome observed in this case is a well‐recognized paraneoplastic condition, reported in 24%–57% of dogs with SCTs, and results from hormonal imbalance due to tumour functionality (Lipowitz et al. 1973; Reif and Brodey 1969; Weaver 1983). Hyperestrogenism secondary to SCTs is a well‐known cause of non‐inflammatory alopecia. The alopecia associated with hyperestrogenism results from oestrogen binding to oestrogen receptor α, which is involved in regulation of the hair follicle cycle. This interaction disrupts the anagen–telogen transition by inducing premature catagen and prolonging the telogen phase, leading to impaired hair growth (Hu et al. 2012). Additional signs of feminization, including gynaecomastia and a pendulous prepuce, are commonly described in this condition. Whereas haematuria and preputial blood discharge have been reported less frequently and are usually associated with underlying prostatic disease (Abalaka and Audu 2024; Pothiappan et al. 2025; Quartuccio et al. 2012; Spackman and Roth 1988). In our clinical case, the ultrasonographic appearance of the prostate was consistent with benign cystic prostatic hyperplasia. It is well recognized that in an older intact male dog, age‐related benign prostatic hyperplasia (BPH) is commonly present. However, severe hyperestrogenism secondary to a functional SCT can markedly alter the androgen‐to‐oestrogen balance, inducing squamous metaplasia of the prostatic epithelium, ductal occlusion and fluid accumulation, thereby exacerbating cystic parenchymal changes (Spackman and Roth 1988).

In the present case, the mild normocytic, normochromic, non‐regenerative anaemia identified was attributed to the documented hyperestrogenism. As reported in the literature, excessive levels of circulating oestrogens in dogs can induce severe myelotoxicity. This condition typically begins with a transient phase of leucocytosis and thrombocytosis, followed by progressive bone marrow hypoplasia or aplasia affecting multiple cell lines (Sontas et al. 2009). In our patient, the serum oestradiol concentration of 119 pg/mL—approximately six to eight times the physiological upper limit—could have exerted a suppressive effect on erythroid activity. Although oestrogen‐induced myelotoxicity can progress to potentially fatal pancytopenia, the timely diagnosis and surgical removal of the neoplasm led to the complete normalization of haematological parameters during the follow‐up period. This confirms that marrow damage, when identified early, may be reversible once the source of hormonal stimulation is eliminated.

In our case, the diagnosis of this condition was essentially clinical and supported by oestrogen serum levels, although preputial cytology and testicular ultrasonography have proven to be very useful, as reported in previous studies (Bigliardi et al. 2019; Cutler et al. 2022). In healthy dogs, age, body weight and breed are associated with specific vaginal epithelial cell population prevalences, while superficial and cornified cells show a negative correlation in the absence of elevated oestrogen levels (Vergou et al. 2023). In dogs with testicular masses, the presence of more than 20% superficial cells in preputial smears was significantly associated with a serum oestradiol concentration > 40 pmol/L (Dreimanis et al. 2012). In addition, the number of superficial cells was significantly increased in preputial smears from dogs with alopecia. This pattern of cellular maturation reflects a pronounced estrogenic influence, supporting a diagnosis of feminization syndrome. The clinical manifestations are strongly linked to a decreased testosterone/oestradiol ratio, and may occur even when absolute oestradiol levels appear within the physiological range due to the production of other oestrogen‐like substances by the tumour (Mischke et al. 2002). In the present case, testosterone levels were not evaluated due to the known episodic and pulsatile secretion of testosterone in male dogs, which results in marked diurnal fluctuations and limits the diagnostic reliability of a single basal measurement (DePalatis et al. 1978). Instead, the diagnosis of functional SCT was robustly supported by the markedly elevated serum oestradiol levels, preputial cytology and characteristic signs of hyperestrogenism (Mischke et al. 2002). Preputial cytology is currently performed only occasionally, mainly for the evaluation of inflammatory and neoplastic conditions. Although further studies are required to assess the reliability of exclusively cornified cells as predictors of hyperestrogenism, the primary aim of the present study was to re‐evaluate this diagnostic approach and highlight the need for its further validation in clinical practice. Finally, histological confirmation and the complete resolution of clinical signs and laboratory abnormalities after castration further supported the diagnosis and our etiopathogenetic hypotheses regarding the observed clinical signs.

## 6 Conclusion

In conclusion, this case supports the inclusion of SCTs among the primary differential diagnoses in older intact male dogs presenting with diffuse dermatological changes, signs of feminization or haematological abnormalities, regardless of breed or the presence of cryptorchidism. The occurrence in a Bernese Mountain Dog with normally descended testes further emphasizes the importance of thorough physical examination in geriatric patients, including careful scrotal palpation and testicular ultrasonography.

The clinical and laboratory findings observed in this dog are consistent with tumour‐associated hyperestrogenism, which contributed to the clinical signs observed. The normalization of haematological parameters and complete resolution of clinical signs following orchiectomy further support the association between hormonal imbalance and systemic manifestations.

In this specific case, preputial cytology served as an interesting observational finding that mirrored the underlying hyperestrogenism. However, as this observation is limited to a single case report, the clinical utility of preputial cytology remains preliminary. While an increased proportion of superficial or cornified cells may offer supportive context alongside hormonal assays and imaging, extensive, controlled prospective studies are strictly required to evaluate its sensitivity, specificity and true diagnostic value.

Overall, early recognition and timely surgical intervention appear crucial to prevent progression towards severe complications such as pancytopenia and to ensure an excellent long‐term prognosis, as demonstrated by complete clinical recovery and full hair regrowth during follow‐up.

## Author Contributions

**Ariu Federica:** conceptualization, investigation, writing – original draft, project administration and supervision. **Burrai Giovanni Pietro:** formal analysis (cytological and histological) and writing – review and editing. **Cubeddu Francesca:** investigation (anaesthesia) and resources. **Masala Gerolamo:** methodology (surgery) and investigation. **Moalli Marzia:** investigation (clinical diagnostic), visualization and writing – review and editing. **Antuofermo Elisabetta:** contributed to data interpretation and manuscript drafting and revision. **Corda Francesca:** investigation (ultrasonography) and writing – review and editing. **Pinna Parpaglia Maria Luisa:** validation, writing – review and editing and supervision. **Corda Andrea:** conceptualization, investigation (ultrasonography), supervision and writing – review and editing.

## Funding

The authors have nothing to report.

## Ethics Statement

This study was conducted in accordance with high standards of veterinary care. The owner of the dog provided written informed consent for the clinical procedures and the publication of this case report. Approved by the Ethics Committee of the University of Sassari, protocol no. 28277 of 10 March 2026.

## Conflicts of Interest

The authors declare no conflicts of interest.

## Data Availability

The data that support the findings of this study are available on request from the corresponding author. The data are not publicly available due to privacy or ethical restrictions.
